# Resilience of cactus pear genotypes in a tropical semi-arid region subject to climatic cultivation restriction

**DOI:** 10.1038/s41598-020-66972-0

**Published:** 2020-06-22

**Authors:** Ricardo Loiola Edvan, Rute Ribeiro Marins Mota, Tairon Pannunzio Dias-Silva, Romilda Rodrigues do Nascimento, Sheila Vilarindo de Sousa, Alex Lopes da Silva, Marcos Jácome de Araújo, Jucilene Silva Araújo

**Affiliations:** 1 Departamento de Zootecnia, Universidade Federal do Piauí, Professora Cinobelina Elvas Campus, Bom Jesus, Piauí Brazil; 2 Instituto Nacional do Semiárido, Campina Grande, Paraíba Brazil

**Keywords:** Plant physiology, Climate-change impacts

## Abstract

The cactus pear has demonstrated productive potential in arid and semi-arid regions due to its photosynthetic process of crassulacean acid metabolism. Thus, this study aimed to evaluate agronomic characteristics and chemical composition of three genotypes of cactus pear at different locations of a tropical semiarid region classified as non-suitable for cactus pear cultivation. A completely randomized design in a factorial arrangement (3 × 7) (three genotypes of cactus pear [Miúda, Baiana (*Nopalea cochenillifera*) and Orelha de Efefante Mexicana - OEM (*Opuntia stricta*)] and seven locations) was used. The climatic conditions characterized an environment that restricts the growth of cactus pear genotypes, mainly due to the air relative humidity values. All morphological characteristics of the cactus pear genotypes were influenced by the interaction genotype x location, with higher expression of the characteristics on the different genotypes under hot semi-arid climate and tropical wet and dry climate. An effect of the interaction genotype x location was observed (*p* < 0.05) on water use efficiency, water accumulation and carrying capacity, where the highest values were observed for genotype Baiana at location 1. Also, there was influence of the interaction genotype x location over the chemical composition of the cactus pear. The cultivation of cactus pear is recommended under restricted climatic conditions in semi-arid tropical regions, especially the genotype Baiana, based on growth factors, biomass production and chemical composition.

## Introduction

Cactus pear has been an important alternative for livestock feeding in arid and semiarid regions, which are characterized by long periods of drought. The lack of rainfall limits the growth of native and cultivated species, such as grasses and legumes of high-water requirement, causing the decrease in biomass and in pasture carrying capacity^1^.

The cactus pear has demonstrated high productive potential in environments with scarce and erratic rainfall. This productive capacity occurs due to its photosynthetic process called Crassulacean Acid Metabolism (CAM), which captures carbon dioxide at night, making the cactus pear efficient in the use of water (100 to 150 kg of water per kg of dry matter), which is about six times more efficient than legumes and almost three times more efficient than grasses^1^. This differentiated photosynthetic mechanism was decisive for the adaptation of this cactus to the hostile climatic conditions of arid and semi-arid regions.

In Brazil, the main cultivated genera are *Opuntia* and *Nopalea*, with emphasis on *Opuntia ficus-indica* (genotypes gigante, redonda and clone IPA-20) and *Nopalea cochenillifera* (genotype doce). This forage plant has high yields of forage biomass, is an excellent source of energy, and is rich in non-fibrous carbohydrates and total digestible nutrients. It presents productivity in a densified crop after two years of planting reaching up to 220 t ha^−1^ of green biomass, depending on the conditions under which the crop is submitted^2^. However, its biomass production and chemical composition are influenced by the growing environment.

Because it is a crop with high adaptive capacity to arid and semi-arid climatic conditions, its cultivation has been carried out without any technical knowledge on the climatic characteristics that favor the growth of the different cactus pear genotypes. For this reason, it is necessary to understand the climatic suitability of genotypes, their agronomic characterization and chemical composition under certain climatic conditions^3^. Thus, we hypothesized that even in semi-arid areas climatically classified as restricted to cactus pear cultivation (as demonstrated in Table 1), it still can present high production of forage biomass and adequate chemical composition, making its cultivation feasible, even under adverse climatic conditions.

**Table 1:** Location, meteorological and aptitude for the cultivation of cactus pear genotypes at the planting locations.

Location	Geographic Coordinates	Altitude (m)	Precipitation (mm)^¥^	T (°C) Min. – Max.^¥^	ARH (%)^¥^	Köppen Classification*	Aptitude^#^
1	Lat. 09°04′28″S and Long. 44°21′31″W	220	905.7	21.1–34.6	42.3	Bsh	Restricted
2	Lat. 09°00′25″S and Long. 44°24′39″W	320	903.9	21.0–34.4	42.1	Bsh	Restricted
3	Lat. 10°07'31″S and Long. 44°57'09″W	400	1,000.4	20.8–34.2	43.0	AW	Restricted
4	Lat. 10°19′32“S and Long. 44°16′86“W	389	958.4	20.7–34.4	43.0	AW	Restricted
5	Lat. 10°02′11″S and Long. 44°18′22″W	350	996.6	20.6–34.4	41.7	Bsh	Restricted
6	Lat. 10°08'12″S and Long. 43°56'55″W	400	935.5	20.4–34.3	42.0	Bsh	Restricted
7	Lat. 09°26′34″S and Long. 45°09′43″W	438	873.5	20.2–34.2	43.3	AW	Restricted

^¥^Data referring to the period of January 2015 to January 2016. Source: http://www.inmet.gov.br/portal/index.php?r=bdmep/bdmep.

*Medeiros *et al*.^4^; ^#^Aptitude for the cultivation of cactus pear regarding temperature and air relative humidity, Lucena *et al*.^3^.

Bsh - Hot semi-arid climate; AW - Tropical savanna climate or tropical wet and dry climate.

Therefore, the present work was conducted with the objective of evaluating the agronomic characteristics and chemical composition of three genotypes of cactus pear in different locations of a tropical semi-arid region classified as restricted to cactus pear cultivation.

## Materials and Methods

### Study location

The experiment was carried out in micro regions belonging to the southern region of the state of Piauí (tropical semi-arid), Brazil, from January 2015 to January 2016.

The experimental design adopted was completely randomized with tem replicates in a factorial scheme (3 × 7). The factors were three genotypes of cactus pear [Miúda, Baiana (*Nopalea cochenillifera* Salm-Dyck) and Orelha de Elefante Mexicana – OEM (*Opuntia stricta* Haw.)] and seven locations (1-Bom Jesus, 2-Currais, 3-Riacho Frio, 4-Curimatá, 5-Júlio Borges, 6-Avelino Lopes and 7-Corrente). The replicates consisted of ten plants for each location x genotype combination, following the recommendations of Donato *et al.*^4^, for the cactus pear crop.

**Figure 1:**
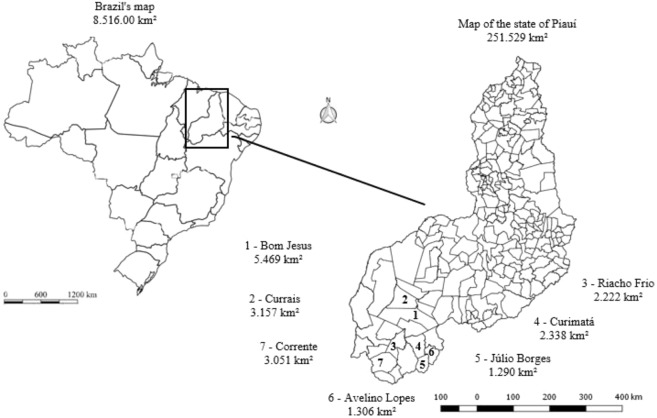
Map with the locations in which the experiments were performed. Source: Author.

The locations where the evaluations were carried out sit in the Chapadas do Extremo Sul Piauiense region, Brazil. Figure 1 shows a map of the state of Piauí, Brazil, highlighting the evaluated locations.

The location and climate characteristics recorded in the micro regions studied are shown in Table 1. The climate of the locations of this region are classified as Aw tropical hot and humid, with summer rains and dry winters (locations 3, 4 and 7) and BSh hot semi-arid, with summer rains and dry winter (locations 1, 2, 5 and 6) according to the Köppen classification, described by Medeiros *et al*. and Alvares *et al*.^5,6^.

The aptitude for the cultivation of cactus pear at each location according to the temperature and air relative humidity is demonstrated in Table 1, as classified by Lucena *et al*.^3^ who consider as Proper (suitable for cultivation), Restricted (limited cultivation) and Improper (outside the proper ranges for the development of the plant), for the cultivation of cactus pear.

The cultivated areas were 135 m² (13.5 × 10 m), planted at a spacing of 1.5 × 0.1 m and with a density of 66,133 plants per ha^−1^, in total 900 plants were cultivated in each location, 300 for each genotype, being evaluated ten plants for each genotype x location combination. Before the implementation of the experiment, soil samples from the 0–20-cm layer were collected from each location for analysis of the chemical composition at the Center of Soil Analysis of the Federal University of the Piauí (UFPI), Bom Jesus, Piauí, Brazil. The soils of the locations were classified as Dystrophic red-yellow Latosols associated with quartz sands. Soil chemical analysis of the seven locations presented values ranging from: 5.2–6.2 of pH in water; 4.2–14.6 mg dm^−3^ of phosphorus (P); 40–84 mg dm^−3^ of potassium (K); 0.3–2.9 cmolc dm^−3^ of calcium (Ca); 0.6–1.3 cmolc dm^−3^ of magnesium (Mg); 0.0–0.6 cmolc dm^−3^ of aluminum (Al); 2.3–3.3 cmolc dm^−3^ of hydrogen + aluminum (H + Al); 1.8–4.3 cmolc dm^−3^ of sum of bases (SB); 0.2–4.3 effective CEC (t); 4.1–7.5 cmolc dm^−3^ CEC at pH 7.0 (T); 30–60% of saturation of bases (V) and 0.3–9.9% of aluminum saturation (M). The physical characteristics of the soil ranged from 150–260 g kg^−1^ of clay, 10–50 g kg^−1^ of silt and 700–810 g kg^−1^ of sand.

### Soil correction and fertilization

Soil correction with dolomite limestone (PRNT 80%) was performed at the locations with SB less than 60%, adding the necessary amount to raise SB up to 60%, thus maintaining SB levels equal in all locations. The base fertilization consisted of 75 kg ha^−1^ of phosphorus in the form of a single superphosphate (18% of P_2_O_5_) and 40 kg ha^−1^ of potassium in the form of potassium chloride (48% of K_2_O). After 30 days of planting, a fertilization with 100 kg ha^−1^ of nitrogen in the form of urea (45% of N) was performed for all locations according to the crop requirement. All fertilization recommendations for cactus pear genotypes were performed considering the crop as demanding.

### Plant samples and measurements

The plants were cut one year after planting using a knife (Tramontina®, 10 inches stainless steel) according to the recommendations of Lima *et al*.^7^, since the production evaluation cycle of the cactus pear is of one year. The cladodes were cut above the main cladode, conserving the mother plant (matrix) to maintain the perenniality of the crop^2^. After cutting, evaluations of the growth, production and chemical composition characteristics of the three genotypes of cactus pear (Miúda, Baiana and Orelha de Elefante Mexicana) were performed. The following morphometric variables were recorded to characterize plant growth: number of cladodes, obtained by counting the cladodes; height of the plant, measured with a measuring tape (100 cm) from the soil surface to the apex of the highest cladode; length (horizontal part) and width (vertical part) of the cladodes, measured in the central region of the cladodes with the same measuring tape (100 cm) which was also used to determine the perimeter of the cladodes. The thickness of the cladodes was measured with a precision digital caliper of 0.05 mm, with all measures taken at the third middle of the cladodes.

During the cutting, the harvested material was weighed in the field, to obtain the total green biomass. Then, a sample of about 2 kg of green matter was taken for laboratory analysis and determination of the dry mass, where it was chopped, weighed and put in a forced air ventilation oven at 65 °C until reaching constant weight (Method INCT-CA G-003/1). The dry samples were then weighed to determine the dry forage biomass and after being ground in a “Thomas Wiley” stationary mill with a 1.0 mm mesh sieve they were stored in containers with covers for laboratory chemical analysis.

The water use efficiency (kg of DM mm^−1^) was estimated by dividing the dry biomass in t ha^−1^ by the amount of rain accumulated during the experimental period. To estimate water accumulation in t ha^−1^, green biomass in t ha^−1^ was multiplied by the percentage of water in the plant, which was calculated by subtracting the dry matter (DM) content from 100, then dividing the result by 1000.

A simulation of the animal carrying capacity was carried out for each of the cactus pear genotypes. For this, a hectare was used to confine sheep for a period of 90 days, and a known dry biomass (t ha^−1^) was used; the sheep were considered to have an average live weight of 25 kg (LW) with an intake of 3% based on LW and 60% of the average daily gain (ADG). The following formula was applied: Animal carrying capacity = (DM t ha^−1^)/(individual intake × 90 days of confinement), where the animal carrying capacity = number of animals.

### Chemical analysis

The determination of the chemical composition was carried out at the Animal Nutrition Laboratory (LANA) of the UFPI, following the methodologies described by Detmann *et al*.^8^. Thus, dry matter (DM) (Method INCT-CA G-003/1), crude protein (CP) (Method INCT-CA N-001/1), ether extract (EE) (Method INCT-CA G-004/1), mineral matter (MM) (Method INCT-CA M-001/1), and neutral detergent fiber (NDF) (Method INCT-CA F-002/1) were determined.

The total carbohydrates (TCHO) and non-fibrous carbohydrates (NFC) were estimated using the equations proposed by Sniffen *et al*.^9^ and Mertens^10^, respectively.

1 $${\rm{TCHO}}=100-( \% {\rm{CP}}+ \% {\rm{EE}}+ \% {\rm{MM}})$$

2 $${\rm{NFC}}=100- \% {\rm{MM}}-{\rm{CP}}- \% {\rm{EE}}- \% {\rm{NDF}}$$

Due to a problem in the storage of samples at locations 2 and 6, the analyses of chemical composition were performed for locations 1, 3, 4, 5 and 7.

### Data analysis

The data were submitted to analysis of variance. The significant interactions were unfolded using the Scott-Knott’s test. All statistical analyses were performed using the software SISVAR version 5.0 and the differences were considered significant for a p-value ≤ 0.05.

## Results

According to Table 1, the climatic conditions of the seven locations ranged from 903.9 to 1000.4 mm year^−1^ of rainfall, 20.2 to 34.6 °C of air temperature and 41.7 to 43.3% of air relative humidity.

The interaction genotype x location affected (*p* < 0.05) all the morphological characteristics of the cactus pear genotypes (Table 2). The genotype Baiana presented thicker cladodes (*p* < 0.001) at locations 1, 2, 5 and 6, which are classified as Bsh climate.

**Table 2:** Morphological characteristics of cactus pear genotypes cultivated in different locations.

Loc^a^	Cactus pear genotypes (Ge)			*P - value*			SEM^b^
	Miúda	OEM^b^	Baiana	Ge	Loc	Ge × Loc	
**Cladode Tickness (mm)**							
1 (Bsh)	12.1bB	8.0cB	16.5aA				
2 (Bsh)	13.0aA	14.8aA	14.6aA	<0.001	<0.001	<0.001	1.23
3 (AW)	9.7aB	8.2aB	9.7aB				
4 (AW)	9.3aB	9.9aB	9.8aB				
5 (Bsh)	11.1bB	12.0bA	18.0aA				
6 (Bsh)	10.4bB	14.3aA	16.9aA				
7 (AW)	15.5aA	7.3bB	9.0bB				
**Cladode Lenth (cm)**							
1 (Bsh)	14.4bA	23.1aB	20.5aC				
2 (Bsh)	17.2bA	24.9aB	25.9aB				
3 (AW)	15.6bA	20.7aC	19.9aC				
4 (AW)	15.7bA	19.9aC	19.5aC	<0.001	<0.001	<0.001	1.29
5 (Bsh)	16.6bA	21.6aC	21.5aC				
6 (Bsh)	14.0cA	62.3aA	42.8bA				
7 (AW)	16.6aA	18.6aC	20.0aC				
**Cladode Width (cm)**							
1 (Bsh)	6.7bB	14.7aB	10.6aB				
2 (Bsh)	7.8bB	17.3aB	13.4aB				
3 (AW)	13.7aA	13.7aB	9.9aB				
4 (AW)	8.8aB	9.7aC	12.6aB	<0.001	<0.001	<0.001	1.48
5 (Bsh)	7.6bB	14.5aB	9.6bB				
6 (Bsh)	17.8bA	24.4aA	19.2bA				
7 (AW)	7.0bB	12.5aC	8.6bB				
**Plant Height (cm)**							
1 (Bsh)	40.8bD	61.1aA	53.1aB				
2 (Bsh)	55.0bC	58.8bA	73.5aA				
3 (AW)	76.5aA	56.7bA	48.0bB				
4 (AW)	51.1aC	44.5aB	49.0aB	<0.001	<0.001	<0.001	3.82
5 (Bsh)	61.1aB	48.0aB	54.6aB				
6 (Bsh)	58.1aC	65.8aA	48.0bB				
7 (AW)	66.5aB	51.5bB	37.5cC				
**Cladode Perimeter (cm)**							
1 (Bsh)	34.4cA	58.1aA	49.4bB				
2 (Bsh)	39.6bA	61.3aA	60.0aA	<0.001	<0.001	<0.001	2.21
3 (AW)	34.9bA	53.2aB	47.8aB				
4 (AW)	35.5bA	47.1aB	47.1aB				
5 (Bsh)	38.9bA	52.7aB	47.7aB				
6 (Bsh)	38.9bA	64.6aA	42.8bB				
7 (AW)	41.0aA	47.5aB	45.5aB				

^a^Loc.: Locations; ^b^OEM: Orelha de Elefante Mexicana; ^b^SEM: Standard Error of the Mean; ^c^CV: coefficient of variation (%); Means followed by different lowercase letters in the rows, statistically differ by the Scott-Knott test (*p* < 0.05); Means followed by different uppercase letters in the columns, statistically differ by the Scott-Knott test (*p* < 0.05). *Significative at *p* < 0.05; ^ns^non-significative at *p* < 0.05.

The length of cladodes in genotype OEM at location 6 was the highest among the genotypes and locations evaluated. Intermediate values for length of cladodes were observed in the genotype Baiana. And the lowest values were observed in the genotype Miúda, which presented no difference between the locations (Table 3).

**Table 3:** Number of cladodes, green and dry forage biomass production of cactus pear genotypes cultivated in different locations.

Loc^a^	Cactus pear genotypes (Ge)			*P-value*			SEM^b^
	Miúda	OEM^a^	Baiana	Ge	Loc	Ge × Loc	
**Number of cladodes (unit.)**							
1 (Bsh)	5.0aC	4.0aA	5.3aB				
2 (Bsh)	5.1aC	5.8aA	6.0aB				
3 (AW)	13.5aA	7.0bA	4.1bB				
4 (AW)	8.5aB	5.0bA	2.8bB	<0.001*	<0.001*	<0.001*	1.10
5 (Bsh)	11.6aA	5.8bA	4.5bB				
6 (Bsh)	14.5aA	5.3cA	8.6bA				
7 (AW)	14.5aA	6.5bA	3.5bB				
**Green Forage Biomass (t ha**^**−1**^ **year**^**−**1^**)**							
1 (Bsh)	46.2cC	103.0bB	266.9aA				
2 (Bsh)	46.4bC	159.2aA	168.2aB	<0.001*	<0.001*	<0.001*	19.3
3 (AW)	134.5aA	94.3aB	45.8bD				
4 (AW)	51.3aC	56.1aC	30.9aD				
5 (Bsh)	98.8aB	106.3aB	56.7aD				
6 (Bsh)	130.0aA	165.4aA	111.0aC				
7 (AW)	72.6aB	73.2aC	46.4bD				
**Dry Forage Biomass (t ha**^**−**1^ **year**^**−**1^**)**							
1 (Bsh)	5.00cB	14.18bA	31.93aA				
2 (Bsh)	4.54bB	17.63aA	19.70aB	<0.001*	<0.001*	<0.001*	0.83
3 (AW)	14.46aA	12.20aA	4.81bD				
4 (AW)	6.49aB	7.88aB	3.58aD				
5 (Bsh)	11.72aA	11.16aA	8.02aC				
6 (Bsh)	12.45aA	15.58aA	10.08aC				
7 (AW)	2.82aB	0.94aC	0.56aD				

^a^Loc.: Locations; ^b^OEM: Orelha de Elefante Mexicana; ^b^SEM: Standard Error of the Mean; ^c^CV: coefficient of variation (%); Means followed by different lowercase letters in the rows, statistically differ by the Scott-Knott test (*p* < 0.05); Means followed by different uppercase letters in the columns, statistically differ by the Scott-Knott test (*p* < 0.05). *Significative at *p* < 0.05; ^ns^non-significative at *p* < 0.05.

The width of cladodes was also the highest for the genotype OEM at location 6, where among the genotypes, OEM presented a development of 5.2 cm higher than the genotype Baiana and, among locations, a growth of 7.1 cm more than at the location 2.

Regarding the height of the plant, genotypes Miúda and Baiana were taller at locations 3 and 2, respectively. While the genotype OEM was taller at location 6.

Genotype OEM presented the highest cladode perimeter value among the genotypes evaluated. For the genotype Miúda, the perimeter of the cladodes was similar in all locations and it presented the lowest values (Table 2). The genotype Baiana had a larger perimeter at location 6.

The green and dry forage biomass production (t ha^−1^), and the number of cladodes were affected by the interaction (*p* < 0.05) between genotypes and locations (Table 3).

The genotype Miúda presented the highest number of cladodes among genotypes at locations 3, 5, 6 and 7. The number of cladodes in genotype OEM did not differ (*p* > 0.05) among locations. The genotype Baiana produced higher green forage biomass and dry forage biomass at the location 6 compared to others genotypes.

The highest production of green and dry forage biomass was observed for genotype Baiana at location 1 (Bsh climate) with 266.9 t ha^−1^ year^−1^ and 31.93 t ha^−1^ year^−1^, respectively. The genotype Miúda presented the highest production at locations 3 and 6 (AW and Bsh climates, respectively) and the genotype OEM at 2 and 6 (Bsh climate). The production of dry biomass presented large variation, ranging from 0.56 to 31.93 t ha^−1^ year^−1^, with lower yields recorded at locations 4 and 7 (AW climate), regardless of the genotype.

There was an effect of the interaction genotype x location (*p* < 0.05) on water use efficiency (WUE), water accumulation (WAC) and carrying capacity (CCAP) in different cactus pear genotypes (Table 4). The genotype Baiana stood out with the highest WUE, WAC and CCAP at location 1 (Bsh climate). On the other hand, location 7 (AW climate) negatively stood out, as the different genotypes presented low WUE, WAC and CCAP.

**Table 4:** Mean values of water use efficiency (WUE), water accumulation (WAC) and carrying capacity (CCAP) of three cactus pear genotypes.

Loc^a^	Cactus pear genotypes (Ge)	OEM^a^	Baiana	*P-value*	Loc	Ge × Loc	SEM^b^
	Miúda			Ge			
**Water Use Efficiency (WUE) (kg DM mm**^**−**1^**)**							
1 (Bsh)	5.0cB	15.0bA	35.0aA				
2 (Bsh)	5.0bB	20.0aA	23.0aB	<0.001*	<0.001*	<0.001*	1.0
3 (AW)	14.0aA	12.0aA	4.0bD				
4 (AW)	6.0aB	7.0aB	3.0aD				
5 (Bsh)	12.0aA	11.0aA	8.0aC				
6 (Bsh)	14.0aA	17.0aA	11.0aC				
7 (AW)	3.0aB	1.0aB	6.0aD				
**Plant Water Accumulation (WAC) (t ha**^**−1**^**)**							
1 (Bsh)	45.0bB	98.0bB	259.0aA				
2 (Bsh)	49.0bB	165.0aA	174.0aB	<0.001*	<0.001*	<0.001*	7.0
3 (AW)	119.0aA	82.0aB	41.0aD				
4 (AW)	45.0aB	48.0aC	27.0aD				
5 (Bsh)	90.0aA	99.0aB	50.0aD				
6 (Bsh)	133.0aA	169.0aA	114.0aC				
7 (AW)	22.0aB	7.0aC	4.0aD				
**Carrying Capacity (CCAP) (sheep ha**^**−**1^**)**							
1 (Bsh)	185.0cB	525.0bA	1,182.0aA				
2 (Bsh)	168.0bB	653.0aA	729.0aB	<0.001*	<0.001*	<0.001*	31
3 (AW)	535.0aA	452.0aA	178.0bD				
4 (AW)	240.0aB	291.0aB	132.0aD				
5 (Bsh)	434.0aA	413.0aA	297.0aC				
6 (Bsh)	461.0aA	577.0aA	373.0aC				
7 (AW)	105.0aB	35.0aC	21.0aD				

^a^Loc.: Locations; ^b^OEM: Orelha de Elefante Mexicana; ^b^SEM: Standard Error of the Mean; ^c^CV: coefficient of variation (%); Means followed by different lowercase letters in the rows, statistically differ by the Scott-Knott test (*p* < 0.05); Means followed by different uppercase letters in the columns, statistically differ by the Scott-Knott test (*p* < 0.05). *Significative at *p* < 0.05; ^ns^non-significative at *p* < 0.05.

There was a significant effect (*p* < 0.05) of the interaction genotype x location on the chemical composition of the cactus pear genotypes (Table 5). The results show a consistent variation between locations and genotypes, except for total carbohydrates which was similar among the genotypes.

**Table 5:** Chemical composition of cactus pear genotypes cultivated at different locations, unfolded means.

Loc^a^	Cactus pear genotypes (Ge)			*P-value*			SEM^b^
	Miúda	OEM^a^	Baiana	Ge	Loc	Ge × Loc	
**Dry Matter (DM) g kg**^**−**1^							
1 (Bsh)	99.7bB	126.5aA	109.3bA				
3 (AW)	99.6bB	119.3aA	97.6bA				
4 (AW)	116.2bA	139.8aA	100.8bA	<0.001*	<0.001*	0.03^*^	2.7
5 (Bsh)	108.5aA	99.9aB	100.8aA				
7 (AW)	113.7aA	119.6aA	111.3aA				
**Crude Protein (CP) g kg**^**−**1^ **DM**							
1 (Bsh)	66.1bB	73.5aB	75.5aB				
3 (AW)	92.3aA	94.9aA	88.4bA	<0.001*	<0.001*	0.001*	0.8
4 (AW)	59.5aC	62.8aC	63.5aC				
5 (Bsh)	66.2bB	73.1aB	72.3aB				
7 (AW)	92.4aA	93.9aA	89.1aA				
**Ether Extract (EE) g kg**^**−**1^ **DM**							
1 (Bsh)	4.1bC	15.0aA	14.0aA				
3 (AW)	14.0aA	13.9aA	12.9aA				
4 (AW)	13.8aA	12.1aA	11.9aA				
5 (Bsh)	10.5aB	12.6aA	10.1aA	<0.001	<0.001*	<0.001*	0.5
7 (AW)	9.6bB	13.2aA	5.7cB				
**Total Carbohydrates (TCHO) g kg**^**−1**^ **DM**							
1 (Bsh)	786.8aA	796.9aA	764.9bA				
3 (AW)	733.3aC	727.3aC	739.8aA				
4 (AW)	788.6aA	788.0aA	769.7aA	0.447^ns^	<0.001*	<0.001*	4.0
5 (Bsh)	765.6aB	759.7aB	775.0aB				
7 (AW)	764.5aB	774.1aA	769.3aA				

^a^Loc.: Locations; ^b^OEM: Orelha de Elefante Mexicana; ^c^SEM: Standard Error of the Mean; ^c^CV: coefficient of variation (%); Means followed by different lowercase letters in the rows, statistically differ by the Scott-Knott test (*p* < 0.05); Means followed by different uppercase letters in the columns, statistically differ by the Scott-Knott test (*p* < 0.05). *significative at *p* < 0.05; ^ns^non-significative at *p* < 0.05.

Dry matter content ranged from 97.6 to 139.8 ± 2.7 g kg^−1^, with the highest value observed in the genotype OEM at location 4. The highest CP contents in all evaluated genotypes were found at locations 3 and 7. Regarding the CP and EE contents, genotype OEM also presented the highest concentrations in its composition with 94.9 ± 0.8 and 15.0 ± 0.5 g kg^−1^, respectively. The highest total carbohydrates content (TCHO) was observed in the genotype OEM at location 1.

There was no significant effect (*p* > 0.05) of the interaction genotype x location on mineral matter (MM), organic matter (OM), neutral detergent fiber (NDF) and non-fibrous carbohydrate (NFC). Genotype OEM presented the lowest MM and highest OM contents, and there was no significant effect for NDF and NFC.

Mineral matter content was higher (*p* < 0.001) and, consequently, OM was lower (*p* < 0.05) at locations 5 and 3 (Table 6). Neutral detergent fiber and NFC were not affected (*p* > 0.05) by the locations.

**Table 6:** Chemical composition of cactus pear genotypes cultivated in different locations.

	MM^c^	OM^d^	NDF^e^	NFC^f^
	g kg^−1^ DM			
**Cactus pear genotypes (Ge)**				
Miúda	146.4A	853.5B	196.3A	571.4A
OEM^a^	137.7B	862.2A	216.6A	552.6A
Baiana	147.4A	852.5B	218.0A	545.7A
**Location (Loc)**				
1 (Bsh)	134.3B	865.7A	207.0A	575.8A
3 (AW)	160.9A	839.0B	197.3A	536.1A
4 (AW)	143.2B	856.7A	191.6A	590.4A
5 (Bsh)	151.5A	848.4B	239.5A	527.3A
7 (AW)	129.3B	870.6A	216.0A	553.2A
Ge	0.058^ns^	0.058^ns^	0.686^ns^	0.657^ns^
Loc	<0.001*	<0.001*	0.703^ns^	0.420^ns^
Ge × Loc	0.054^ns^	0.054^ns^	0.134^ns^	0.139^ns^
SEM^b^	3.89	3.89	25.4	26.3

^a^OEM: Orelha de Elefante Mexicana; ^b^SEM: Standard Error of the Mean; ^c^MM: Mineral Matter; ^d^OM: Organic Matter; ^e^NDF: Neutral Detergent Fiber; ^f^NFC: Non-Fibrous Carbohydrates. Means followed by different lowercase letters in the rows, statistically differ by the Scott-Knott test (*p* < 0.05); Means followed by different uppercase letters in the columns, statistically differ by the Scott-Knott test (*p* < 0.05). *Significative at *p* < 0.05; ^ns^non-significative at *p* < 0.05.

## Discussion

The mean temperature of the seven locations during the year when the experiment was carried out was within the climatic average obtained by Medeiros *et al*.^5^ in a 30-year survey (1960 to 1990) with an average temperature of 26.1 °C for the seven locations, the rainfall observed during the experiment was higher while the air relative humidity was lower than the observed in Medeiros *et al*.^5^, with 848 mm year^−1^ for rainfall and 64% of air relative humidity. The climatic conditions, especially regarding to temperature and rainfall, were adequate for the cactus pear, being an important factor for the development of agronomic characteristics of the genotypes, mainly plant’s water accumulation capacity and water use efficiency. However, the overall average of air relative humidity was at the limit (≥40%) of what is considered adequate for the growth of cactus pear. It is worth mentioning that in some days during the execution of the experiment, air relative humidity values lower than 20% were recorded in all locations.

According to the results obtained in the soil analysis of the 7 locations, the same amount of fertilizer was used following the recommendations for cactus pear crop in the locations, and the little difference between the chemical and physical characteristics of the soils despite the distance between the locations is due to the high amount of sand and low soil fertility. It should be emphasized that in the choice of planting areas in the different locations, we opted for sites with similar soil characteristics, since the objective of the research was to evaluate the planting restriction of cactus pear in different locations in regard to the climate.

Based on the results obtained, each genotype adapted and responded better to the edaphoclimatic characteristics of the different locations, where the genotype Baiana presented better adaptation to locations 1, 2, 5 and 6 (Bsh climate), the genotype OEM presented better development at location 6 (Bsh climate) and the genotype Miúda presented good response on number of cladodes at locations 3, 5, 6. Thus, this study is of great importance for semi-arid regions, mainly because it is a region constituted by locations of great territorial extensions with different climatic characteristics that directly affect the agronomic characteristics of the genotypes.

An important morphological characteristic of the genotype Baiana is the larger thickness of the cladodes. Although from the same species and same genus (*Nopalea*) of the genotype Miúda, they have different morphological characteristics, and its cladodes have medium elliptic shape and considerable thickness. The averages of temperature, ARH and annual precipitation during the experimental period at location 5 were favorable to the growth of this genotype, which is more demanding in climatic conditions than the genotype OEM.

Cactus pear genotypes from genera *Nopalea* and *Opuntia* present different morphological characterization of cladodes that are influenced by edaphoclimatic conditions. Studying how these characteristics relate favors the understanding of how the plant responds under different environmental conditions (soil and climatic conditions responses). Plants with cladodes of smaller dimensions can distribute their cladodes with vertical growth, shaping plants with greater height and smaller width, such as the genotype Miúda, which presented more cladodes, an inherent characteristic of this genotype [production of smaller cladodes, however, in a higher amount]^11,12^, which implies an increase in planting areas, due to crop densification^13,14^. On the other hand, plants with larger cladodes can invest in lateral growth, due to their structural form^15^, as it was verified in the genotype OEM at location 6. Similarly, the superior width of cladodes in this genotype in the conditions mentioned above shows that these structural characteristics may be associated to the location of cultivation and to the genus (*Opuntia*), which has broad cladodes and low ratio between the length/width of the cladodes^16^.

Research^17,18^ has shown that plant height and width characteristics directly influence the production of green and dry forage biomass. Thus, plants with greater height can be cultivated with smaller spacing, as the growing size does not limit the development of other plants, increasing productivity and contributing to the rational management in the exploitation of this cactus. On the other hand, for genotypes that present larger plant widths, cultivation with greater spacing between plants is recommended, due to the competition for water, light and nutrients, as well as facilitating crop treatment and harvesting^11^. However, it is worth mentioning that the morphological and chemical characteristics of the plant are also related to the edaphoclimatic characteristics of the location of cultivation and not only to the genotype. Thus, the genotype x soil x climate interaction determines the canopy structure of the cactus pear^19^.

The higher production of green forage biomass and, consequently, of dry forage biomass by the genotype Baiana observed at location 1 is related to some morphological characteristics such as the thickness and perimeter of the cladodes, a fact that favored a better water use efficiency and water accumulation by this genotype (Table 4). These factors are preponderant for a broad expression of the genetic potential of the genotype. Thus, from the point of view of animal production in semi-arid regions in the period of low availability of native or cultivated forage, the use of the genus Nopalea is suggested, especially the genotype Baiana, due to its high biomass production and, consequently, greater carrying capacity.

The opposite occurred with the genotype Miúda, which is considered one of the genotypes that shows higher GFB yield, due to the higher amounts of cladodes produced by the plant. However, considering the smaller size of this genotype and that the spacing between plants was the same for all of the evaluated genotypes, one can then use the strategy of planting according to the genotype to be used. This fact was observed in a study by Lima *et al*.^20^ who obtained 44.7 t ha^−1^ year^−1^ of dry biomass with a density of 80,000 plants ha^−1^. In the present study the planting density was 67,000 plants ha^−1^. For the cactus pear Miúda, the planting is dimensioned with a smaller spacing between plants, increasing the number of plants, thus obtaining a greater number of cladodes and, consequently, a greater production of forage biomass.

The locations 4 and 7 obtained, regardless of the genotypes, production of dry biomass <10 t ha^−1^ year^−1^. Both locations have AW climate classification, and this type of climate is classified by Medeiros *et al*.^4^ with rains in the summer and dry in winter, when the air relative humidity usually reaches values below 40%. The locations 4 and 7 were confirmed as locations restricted to the cultivation of cactus pear, however, more evaluations are needed over time before condemning the cultivation of this crop in these locations. On the other hand, in the locations 1, 2, 3, 5 and 6 the genotypes presented higher growth and production, under same climate conditions (regions of cultivation restriction). This occurs due to their CAM metabolism, which acts by capturing carbon dioxide at night, as well as having low transpiration rate and closure of the stomata during the day, making these plants highly efficient in the use of water compared to C3 and C4 metabolism plants^21^. Such mechanism makes these plants more efficient in the use of water^2,22^. This means that CAM plants lose from 50 to 100 g of water per gram of CO_2_ fixed, while C3 and C4 plants lose from 400 to 500 g and from 250 to 300 g of water per gram of CO_2_ fixed, respectively.

Thus, the adaptive capacity of the cactus pear to dry environments is highlighted^1,23^, since it combines the production of green biomass with the high moisture content in its composition, as well as non-fibrous carbohydrates, making it possible for the farmers to use this forage not only as a possible alternative food for the herds, but as an available source throughout the year. Therefore, the identification and selection of the genotype that best suits the given micro region, directly influences the optimization of resources (greater efficiency of land and water use), aiming to obtain high yields of biomass and better chemical composition of cactus pear^23,24^.

Cactus pear, regardless of genotype, should be included as a source of roughage in animal feeding. It should be noted, however, that the cactus pear presented a low percentage of dry matter (Table 5) regardless of the genotype and location, and this may compromise the rumen functions when offered in inadequate amounts, causing digestive disturbances in the animal.

The CP content observed in the present study were high for all genotypes and locations when compared to the study of Cavalcante *et al*.^25^ who observed CP of 43.1 g kg^−1^ DM for the genotype Miúda. These high CP values were probably due to the nitrogen fertilization of 100 kg of N ha^−1^. When herd productive efficiency is sought using this source of forage, it is mandatory to use another protein source for the animals, in order to adapt the protein:carbohydrate ratio, maximizing the efficiency of dietary nutrients utilization, and promoting microbial growth and efficiency of microbial synthesis^26^.

Regardless of the genus, cactus pear presented considerable non-fibrous carbohydrates contents (there was no difference between genotypes and locations). The low levels of NDF, such as those found in this study (values lower than 191.6 g kg^−1^ DM), decrease the total chewing time, reducing saliva secretion, which is rich in buffering agents and essential for maintaining ruminal physiology^27^.

According to Dubeux Jr *et al*.^28^ the cactus pear presents considerable contents of total carbohydrates, non-fibrous carbohydrates (NFC), non-structural carbohydrates and mineral matter. Although there was no difference for the genotypes and locations in the present study, the cactus pear presented values of NFC higher than 527.3 g kg^−1^ DM. The chemical composition of the cactus pear highly varies according to the cultivated genus, the age of the articles of each cladode, the season of the year, the crop treatments and the edaphoclimatic conditions of the location of cultivation^2^.

## Conclusions

The resilience of the cactus pear in different semi-arid tropical environments proves the potential of this plant as a source of roughage for animal feed.

The genotypes of cactus pear exhibited different productive responses in relation to the locations of cultivation, requiring further studies to assess the potential of each genotype for specific locations.

The genotype Baiana has the greatest forage production potential among the genotypes for the different locations in the semi-arid tropical climate evaluated.

## References

[1] Nunes VX, Nunes NX, Londe LN, Oliveira CG, Rocha SS (2017). Physico-chemical characterization of prickly pear (Opunicia Ficus indica) in the semi-arid region of Bahia State, Brazil. Afr. J. Agric. Res..

[2] Nefzaoui A (2009). Cactus: A Crop to Meet the Challenges of Climate Change in Dry Areas. Ann. Arid Zone..

[3] Lucena DB, Medeiros RM, Saboya LMF, Nascimento PL (2016). Aptidão e zoneamento agroclimático da palma forrageira para o estado do Piauí. Rev. Bras.Agric. Irrig.

[4] Donato PE (2014). Morfometria e rendimento da palma forrageira ‘Gigante’sob diferentes espaçamentos e doses de adubação orgânica. Rev. Bras. Cienc. Agrar..

[5] Medeiros RM, Santos DC, Sousa FAZ, Gomes Filho MF (2013). Análise Climatológica, Classificação Climática e Variabilidade do Balanço Hídrico Climatológico na Bacia do Rio Uruçui Preto, PI. R. Bras. Geog. Fís.

[6] Alvares CA, Stape JL, Sentelhas PC, Goncalves JLM, Sparovek G (2013). Köppen’s climate classification map for Brazil. Meteorol. J.

[7] Lima GFC (2013). Effect of different cutting intensities on morphological characteristics and productivity of irrigated Nopalea forage cactus. Acta Hortic..

[8] Detmann, E. *et al*. Métodos para Análise de Alimentos - INCT - Ciência Animal. 1 ed. Visconde do Rio Branco: Suprema, 214p. (2012).

[9] Sniffen CJ, O’Connor JD, Van Soest PJ, Fox DG, Russell JB (1992). A net carbohydrate and protein system for evaluating cattle diets: II. Carbohydrate and protein availability. J. Anim. Sci..

[10] Mertens DR (1997). Creating a system for meeting the fiber requirements of dairy cows. J. Dairy Sci..

[11] Silva JÁ, Donato SLR, Donato PER, Souza ES, Padilha Júnior MC (2016). & Silva Junior. AA. Yield and vegetative growth of cactus pear at different spacings and under chemical fertilizations. Rev. Bras. eng. agric.

[12] Ewela JJ, Mazzarino MJ (2008). Competition from below for light and nutrients shifts productivity among tropical species. Pnas.

[13] Gebreegziabher Z, Tsegay BA (2015). Efficacy of cactus pear (*Opuntia ficus-indica*) varieties as a source of food and feed in endamehoni district, Northern Ethiopia. Afr. J. Food Agric. Nutr. Dev..

[14] Amorim PL, Martuscello JA, Araújo Filho JT, Cunha DNFV, Jank L (2015). Morphological and productive characterization of forage cactus varieties. Rev. Caatinga.

[15] Neder DG, Costa FR, Edvan RL, Souto Filho LT (2013). Correlations and path analysis of morphological and yield traits of cactus pear accessions. Crop. Breed Appl. Biotechnol..

[16] Siqueira MCB (2017). Optimizing the use of spineless cactus in the diets of cattle: Total and partial digestibility, fiber dynamics and ruminal parameters. Anim. Feed. Sci. Technol..

[17] Rahul D, Singh JP, Singh T, Dayal D (2018). Effect of Shade Levels on Growth, and Biomass Production of Cactus (*Opuntiaficus-india* (L.) Mill.). Inter. J. Cur. Microb. Appl. Scien..

[18] López US, Nieto CAR, Rangel PP, Real D (2018). Yield of forage, grain and biomass in eight hybrids of maize with different sowing dates and environmental conditions. R. Caatinga.

[19] Barbosa ML (2018). The influence of cladode morphology on the canopy formation of forage cactus plants. Rev. Ceres.

[20] Lima GFC, Rego MMT, Dantas FDG, Lôbo RMB, Aguiar EM (2016). Morphological characteristics and forage productivity of irrigated cactus pear under different cutting intensities. R. Caatinga.

[21] Han H, Felker P (1997). Field validation of water-use efficiency of the CAM plant *Opuntia ellisiana* in south Texas. J. Arid Environ.

[22] Andrade JAS (2015). Production of Peanut Intercropped with Forage Palm in Pernambuco State, Brazil. Am. J. Plant Sci.

[23] Goldstein G, Ortega JK, Nerd A, Nobel PS (1991). Diet patterns of water potential components for the Crassulacean acid metabolism plant *Opuntia ficus-indica* when well-watered or droughted. Plant Physiol..

[24] Sales AT (2009). Adaptation potential of cactus pear to soil and climatic conditions of the Semi-Arid in Paraiba State, Brazil. Acta Hortic.

[25] Cavalcante LAD, Santos GRA, Silva LM, Fagundes JL, Silva MA (2014). Respostas de genótipos de palma forrageira a diferentes densidades de cultivo. Pesq. Agropec. Trop.

[26] Russell JB, O’Connor JD, Fox DG, Van Soest PJ, Sniffen CJ (1992). A net carbohydrate and protein system for evaluating cattle diets. 1. Ruminal fermentation. J. Anim. Sci..

[27] Vilela MS (2010). Evaluation of feeding supply and forage cactus processing for lactation cows. R. Bras. Zootec.

[28] Dubeux JR (2006). JCB, Santos, MVF & Lira, MA. Productivity of Opuntia ficus-indica (L.) Miller under different N and P fertilization and plant population in north-east Brazil. J. Arid Environ..

